# Endocannabinoids and the Gut-Brain Control of Food Intake and Obesity

**DOI:** 10.3390/nu13041214

**Published:** 2021-04-07

**Authors:** Nicholas V. DiPatrizio

**Affiliations:** Division of Biomedical Sciences, School of Medicine, University of California Riverside, Riverside, CA 92521, USA; ndipatri@medsch.ucr.edu; Tel.: +1-951-827-7252

**Keywords:** endocannabinoid, CB_1_ receptor, gut-brain, intestine, food intake, reward

## Abstract

Gut-brain signaling controls food intake and energy homeostasis, and its activity is thought to be dysregulated in obesity. We will explore new studies that suggest the endocannabinoid (eCB) system in the upper gastrointestinal tract plays an important role in controlling gut-brain neurotransmission carried by the vagus nerve and the intake of palatable food and other reinforcers. A focus will be on studies that reveal both indirect and direct interactions between eCB signaling and vagal afferent neurons. These investigations identify (*i*) an indirect mechanism that controls nutrient-induced release of peptides from the gut epithelium that directly interact with corresponding receptors on vagal afferent neurons, and (*ii*) a direct mechanism via interactions between eCBs and cannabinoid receptors expressed on vagal afferent neurons. Moreover, the impact of diet-induced obesity on these pathways will be considered.

## 1. Introduction

Gut-brain signaling plays an integral role in food intake, energy homeostasis, and possibly reward [1,2,3]. Our understanding of the biochemical and molecular pathways involved in these processes and their dysregulation in obesity, however, remains incomplete. Several signals, including gut-derived peptides, have been identified that control neurotransmission from peripheral organs to the brain (see for comprehensive review [4]). These include cholecystokinin (CCK), which is released from subpopulations of enteroendocrine cells in the upper small-intestinal epithelium in response to the presence of nutrients in the lumen and controls food intake and meal size by activating the afferent vagus nerve [1,5,6,7,8,9]. Recent studies in mice suggest that specialized enteroendocrine cells in the intestinal epithelium, termed “neuropods”, form functional synapses with gastric afferent vagal fibers and participate in the transduction of signals from food to neural signals carried by vagal afferent neurons to the brain [10]. Neuropods sense nutrients on their luminal side and, in turn, release glutamate and CCK in a coordinated manner that induces rapid or prolonged firing of vagal afferent neurons, respectively [9]. These results highlight neuropods as a key cellular mechanism in nutrient sensing and associated gut-brain signaling. Other studies suggest that vagal afferent neurotransmission recruits brain reward circuits and may participate in food reward [11,12,13,14]. For example, optogenetic activation of right gastric vagal afferent neurons increased (i) dopamine release in central reward pathways, (ii) operant responses associated with self-stimulation of brain reward neurons, and (iii) conditioned flavor and place preferences [11]. Specific biochemical and molecular signaling pathways that control these functions, however, remain unclear.

**Figure 1:**
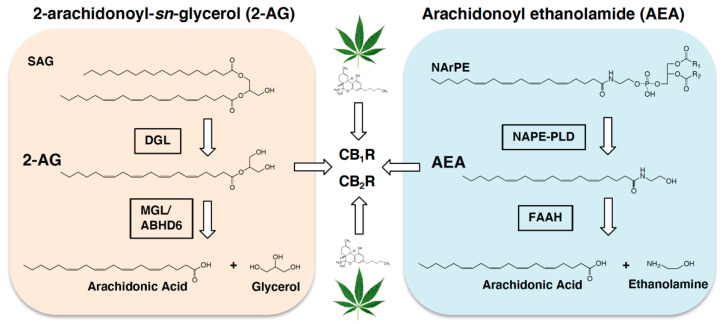
Endocannabinoid metabolic pathways. Biosynthesis of the endocannabinoid, 2-arachidonoyl-*sn*-glycerol (2-AG), is facilitated by diacylglycerol lipase- (DGL) dependent hydrolysis of the 2-AG precursor, 1,stearoyl,2-arachidonoyl-*sn*-glycerol (SAG). 2-AG is degraded by monoacylglycerol lipase (MGL), and to a lesser degree by alpha-beta-hydrolase domain 6 (ABHD6), into arachidonic acid and glycerol. Biosynthesis of the fatty acid ethanolamide, arachidonoyl ethanolamide (AEA, anandamide), is controlled by *N*-acylphosphatidylethanolamide phospholipase D- (NAPE-PLD) dependent hydrolysis of the AEA precursor, *N*-arachidonoylphosphatidylethanolamide (NArPE). AEA is degraded by fatty acid amide hydrolase (FAAH) into arachidonic acid and ethanolamine. 2-AG and AEA activate cannabinoid subtype-1 receptors (CB_1_R) and cannabinoid subtype-2 receptors (CB_2_R) in cells throughout the body (see for review [42,43]). Alternate endocannabinoid metabolic pathways have also been suggested. The primary intoxicating chemical in the cannabis plant, ∆^9^ tetrahydrocannabinol (THC, represented by the green leaf and corresponding THC molecule), hijacks the endocannabioid system and activates cannabinoid receptors in cells throughout the body.

The endocannabinoid (eCB) system is a lipid-derived signaling pathway that controls food intake, energy homeostasis, and reward, and is hijacked by chemicals in the cannabis plant [15,16,17,18,19] (see Figure 1). In general, activating the eCB system increases food intake [20] and inhibiting its activity reduces food intake [21]. The eCB system is located throughout the brain and plays an important role in these functions; however, mounting evidence also suggests that the eCB system in peripheral organs, including the small-intestinal epithelium, serves an integral role [22,23,24,25,26,27,28,29,30,31,32,33,34,35,36]. Indeed, pharmacological blockade of peripheral cannabinoid subtype-1 receptors (CB_1_Rs) reduces food intake and improves metabolic dysfunction associated with obesity in rodents similarly to brain-penetrant CB_1_R antagonists [21,23,24,27,28,29,30,37]. These studies highlight the peripheral eCB system as a possible target for safe anti-obesity agents that are devoid of psychiatric side-effects associated with drugs that access CB_1_Rs in the brain (e.g., rimonabant [38]).

The eCB system in the rodent small-intestinal epithelium becomes activated (*i*) during oral exposure to dietary fats [23,39], (*ii*) during a fast [22,24,40], and (*iii*) after chronic exposure to obesogenic diets [25,40,41]. Moreover, pharmacological inhibition of peripheral CB_1_Rs blocked (*i*) cephalic-phase consumption of dietary fats in rats [23,39], *(ii)* refeeding after a fast in rats [40], (*iii*) hyperphagia associated with western diet-induced obesity in mice [25,41], and (*iv*) restored nutrient-induced secretion of satiation peptides in western diet-induced obese mice. These studies suggest a critical role for eCB signaling in the gut in the intake of palatable foods. We will review recent experiments that expand our understanding of roles for the eCB system in the gut in gut-brain neurotransmission associated with food intake, energy homeostasis, and reward. An emphasis will be on studies that reveal both indirect and direct mechanisms of control for CB_1_Rs over gut-brain signaling and dysregulation of these pathways in rodent models of diet-induced obesity.

## 2. Gut-Brain Endocannabinoid Signaling Controls Intake of Palatable Foods

The eCB system is expressed in cells throughout all organs in the body and is comprised of lipid-derived signaling molecules including the primary eCBs, 2-arachidonoyl-*sn*-glycerol (2-AG) and arachidonoyl ethanolamide (anandamide), their metabolic enzymes, and cannabinoid receptor sub-type 1 (CB_1_R), cannabinoid receptor sub-type-2 (CB_2_Rs), and possibly others [42,43] (see Figure 1). The eCB system in the brain is extensively studied for its roles in controlling the intake and reward value of palatable food [44,45,46,47,48,49,50,51,52,53,54,55,56,57,58,59,60]. In addition to central sites, recent evidence suggests that the eCB system located in cells lining the intestinal epithelium is an integral component of a gut-brain axis that controls the intake of palatable foods [61]. For example, a sham-feeding protocol in rats was utilized to test if eCB signaling in the gut is associated with positive reinforcement that drives intake of food based on its orosensory properties [23]. During sham feeding, rats are allowed to freely consume a liquid diet that drains from a surgically-implanted, reversible, cannulae in the stomach before it reaches the small intestine. Therefore, sham feeding enables isolation of the cephalic phase of food intake and effectively eliminates post-ingestive consequences of food intake. [62]. Separate groups of rats were given access for 30 min to a fixed amount of dietary fats (corn oil emulsion), sucrose, or protein, and levels of 2-AG and anandamide were measured in the upper small-intestinal epithelium by liquid chromatography/mass spectrometry [23]. Tasting dietary fats—but not sucrose or protein—triggered production of eCBs in the upper small-intestinal epithelium, but not in other peripheral organs tested (i.e., tongue, stomach, ileum, pancreas, liver) or in micropunches obtained from brain regions associated with food intake and reward (i.e., ventral striatum, dorsal striatum, lateral regions of hypothalamus, medial regions of the hypothalamus, pontine parabrachial nucleus, or cerebellum). This effect was also specific for mono- and di-unsaturated fats (oleic acid and linoleic acid), but not saturated (stearic acid) or polyunsaturated fats (linolenic acid) [39]. Moreover, production of eCBs in the small-intestinal epithelium was absent in sham feeding rats that received full subdiaphragmatic vagotomy, which suggests that efferent vagal signaling participates in the biosynthesis of eCBs. Furthermore, intra-duodenal administration of a low-dose cannabinoid receptor subtype-1 (CB_1_R) inverse agonist or a peripherally-restricted CB_1_R antagonist blocked sham feeding of fats. Collectively, these studies suggest that tasting dietary fats recruits an eCB mechanism in the gut that provides positive feedback to the brain and promotes intake of fatty foods.

The aforementioned studies utilized pharmacological, biochemical, and behavioral approaches to identify roles for peripheral CB_1_Rs in the intake of palatable food. At the time of these studies, however, appropriate tools were not available to directly ask if CB_1_Rs in the intestinal epithelium are required in these processes. To test the necessity for CB_1_Rs in the intestinal epithelium in the intake of palatable foods, we developed transgenic mice (Cnr1^tm1^.^1 mrl^/Vil-CRE ERT2) that are conditionally deficient in CB_1_Rs in the intestinal epithelium (referred to as IntCB_1_-/-mice) [63]. Mice were maintained on standard rodent chow low in fats and sugars, then given access for the first time to a palatable western-style diet high in fats and sugars (Research Diets D12079B; 40% kcals from fats and 43% from carbohydrates [64]), and preferences for western diet were measured. This specific western diet was chosen due to its macronutrient composition that more closely matches the human diet (35% kcals from fat and 47% kcals from carbohydrate [46]) when compared to other obesogenic diets routinely used in rodent studies (e.g., Research Diets D12492; 60% kcals from fat and low levels of carbohydrates). Control mice with functional CB_1_Rs in the intestinal epithelium displayed large preferences for western diet when compared to chow, with over 90% of total kilocalories consumed from western diet over the testing period. In contrast to controls, preferences for western diet were reduced for up to 12 h in IntCB_1_-/- mice. These results provide direct evidence that CB_1_Rs in the murine intestinal epithelium are required for acute preferences for palatable foods.

Similar to rodents, humans prefer fatty and sweet foods when given a choice [65], and their consumption is associated with elevated levels of eCBs in blood [66]. Moreover, levels of eCBs are increased in blood in both human and rodent obesity [25,67,68,69,70,71,72,73,74,75,76,77,78,79,80]; however, the impact that circulating eCBs may have on gut-brain function associated with food intake, dietary preferences, and obesity is unknown. Nonetheless, it is plausible that circulating eCBs act as a humoral signal that interacts with cannabinoid receptors along the gut-brain axis to facilitate these processes.

## 3. Endocannabinoids and Gut-Brain Neurotransmission: Indirect Mechanisms

**Figure 2:**
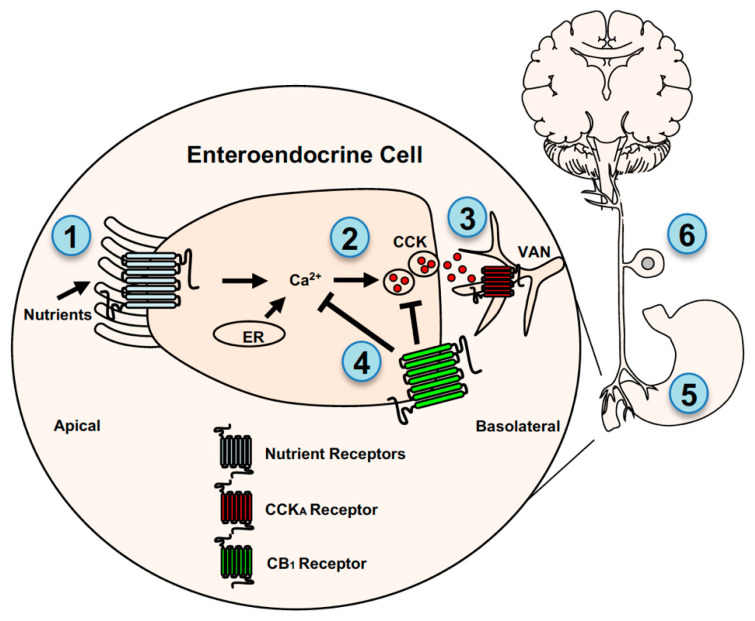
Endocannabinoid system control of gut-brain signaling. CB_1_Rs are located on enteroendocrine cells in the small-intestinal epithelium, stomach cells, and vagal afferent neurons (VANs) where they indirectly and directly control gut-brain neurotransmission. CB_1_Rs are thought to indirectly interact with VANs by a mechanism that includes controlling nutrient-induced secretion of the satiation peptide, cholecystokinin (CCK), from enteroendocrine cells. Nutrients, including fatty acids and glucose/sweeteners, are sensed by mechanisms that may include several distinct G-protein coupled receptors (GPRCs) located on the apical and/or basolateral membrane of enteroendocrine cells (**1**), which triggers calcium-dependent secretion of CCK (**2**) and other signaling molecules. Glucose sensing is also mediated by a mechanism that requires sodium-glucose linked transporter 1 located on the apical membrane of enteroendocrine cells. CCK activates adjacent CCK_A_ receptors on VAN fibers (**3**). Levels of eCBs are elevated in the small-intestinal epithelium in rodent models of diet-induced obesity (see [25]), and their increased activity at local CB_1_Rs blocks nutrient-induced secretion of CCK (**4**) by a mechanism that is unclear but may include inhibition of Ca^2+^-mediated vesicular release of CCK (see [41]). Pharmacological inhibition of peripheral CB_1_Rs in diet-induced obese mice blocked overeating and restored the ability for nutrients to induce CCK release. Studies also suggest that CB_1_Rs on stomach cells (see [81]) may also indirectly interact with VANs by controlling the formation of ghrelin, which can activate ghrelin receptors on VANs (5). Together these studies highlight an indirect mechanism for CB1R-mediated control of gut-brain signaling. CB1Rs may also directly control activity of VANs (6). CB1Rs are expressed in VANs and their expression is affected by feeding status, pharmacological administration of CCK and ghrelin, and diet-induced obesity (see [82,83,84,85,86,87]). Moreover, recent studies suggest that CB1Rs control mechanosensitivity of VANs, which may be dysregulated in diet-induced obesity (see [86,88,89]). ER = endoplasmic reticulum.

Mounting evidence suggests that eCB signaling in the periphery controls food intake by mechanisms that include both indirect and direct interactions with the afferent vagus nerve (see Figure 2). We will first review evidence of an indirect mechanism for CB_1_Rs in the control of gut-brain signaling and its possible dysregulation in diet-induced obesity.

### 3.1. Interactions with Satiation Signaling Pathways

Recent studies in mice suggest that CB_1_Rs in cells lining the small-intestinal epithelium control food intake by blocking nutrient-induced secretion of the satiation peptide, cholecystokinin (CCK), which leads to increased caloric intake and meal size under conditions of heightened local eCB tone (e.g., diet-induced obesity) [25,41]. Upon arrival of nutrients in the small-intestinal lumen, CCK is released from subpopulations of enteroendocrine cells (i.e., I cells) [4,9,81,82] and controls meal size and satiation by directly activating CCK_A_ receptors on vagal afferent neurons [1,5,6,7,8,9] and possibly in the brain [90,91]. Immunoreactivity for CB_1_Rs was found on CCK-containing cells in the upper small-intestinal epithelium in a CCK-reporter mouse that expresses eGFP selectively in these cells [C57BL/6-Tg(Cck-EGFP)2Mirn/J] [41]. CCK-eGFP cells were then isolated by fluorescence-activated cell sorting (FACS) and expression of messenger RNA (mRNA) for components of the eCB system, including CB_1_Rs (Cnr1), was analyzed. CCK-eGFP-positive cells were enriched with mRNA for CB_1_Rs when compared to CCK-eGFP-negative cells, which confirms earlier reports of expression of mRNA for CB_1_Rs in I cells in another CCK-reporter mouse line [92]. We next asked if pharmacological activation of CB_1_Rs with the general cannabinoid receptor agonist, WIN 55,212-2, impacts nutrient-induced release of the bioactive form of CCK, CCK-8. Circulating levels of CCK-8 were increased within 30-min following oral gavage of corn oil, an effect that was completely reversed by pretreatment with WIN 55,212-2. The inhibitory effects of WIN 55,212-2 on corn oil-induced elevations in CCK-8 in blood were blocked by the peripherally-restricted neutral CB_1_R antagonist, AM6545, which highlights a role for peripheral CB_1_Rs in this response.

The study described above was performed in lean mice fed a low-fat and low-sugar diet, which express low levels of eCBs in the small-intestinal epithelium. Diet-induced obesity is associated with high levels of eCBs in the small-intestinal epithelium, [25,36,40,78], and pharmacological inhibition of this heightened eCB activity at peripheral CB_1_Rs blocked overeating resulting from increased meal size and daily caloric intake [25]. These experiments suggest that elevated eCB tone in the small-intestinal epithelium drives the overconsumption of high-energy foods and promotes obesity; however, the mechanism(s) in this response were unclear. Therefore, we tested the hypothesis that heightened eCB signaling at CB_1_Rs in the small-intestinal epithelium in our mouse model of western diet-induced obesity drives overeating by blocking nutrient-induced release of CCK-8. Mice were maintained for 60 days on western diet (Research Diets D12079B), which is a time when levels of eCBs are elevated in the intestinal epithelium. Oral gavage of corn oil increased levels of CCK-8 in blood in lean mice with low levels of eCBs in the intestinal epithelium. In contrast to lean mice, corn oil failed to increase levels of CCK-8 in blood in mice fed a western diet for 60 days; however, pretreatment with the peripherally-restricted CB_1_R antagonist, AM6545, restored the ability for nutrients to increase levels of CCK-8 in blood. These results suggest that under conditions of heightened eCB activity at CB_1_Rs in the small-intestinal epithelium (i.e., diet-induced obesity), CCK-8 release is inhibited, which leads to delayed satiation and overeating. Indeed, inhibition of peripheral CB_1_Rs with AM6545 in obese mice attenuated overeating associated with increased meal size and total caloric intake. Moreover, the hypophagic effects of AM6545 were reversed by pretreatment with a low-dose of the CCK_A_ receptor antagonist, devazepide, which suggests that acute hypophagic effects AM6545 occurs by a mechanism that includes restoring nutrient-induced satiation signaling. Collectively, these studies indicate a key inhibitory role for CB_1_Rs in the small-intestinal epithelium in nutrient-induced secretion of satiation peptides. Thus, CB_1_Rs in the intestinal epithelium are thought to indirectly control gut-brain neurotransmission via regulating the release of gut-derived peptides that directly interact with the vagal afferent neurons (see Figure 2). Furthermore, these processes become dysregulated in diet-induced obesity, which leads to overeating and possibly obesity. Future studies will be important to elucidate (*i*) specific intracellular signaling pathways (e.g., inhibition of calcium channels) in enteroendocrine cells that link eCB signaling at local CB_1_Rs with blockade of secretion of satiation peptides, (*ii*) the impact of eCB activity at CB_1_Rs in the intestinal epithelium on activity of gastric vagal afferent neurons, and (*iii*) the impact that this signaling has on recruitment of brain circuits associated with food reward [11,93].

### 3.2. Interactions with Hunger Signaling Pathways

Recent studies suggest that CB_1_Rs in stomach cells influence alcohol intake and preference in mice by controlling local formation of the bioactive appetite-stimulating hormone, ghrelin [81], which directly interacts with growth hormone secretagogue receptor (GHS-R1a) on vagal afferent neurons and the brain (see Figure 2) [82,94,95]. Godlewski and colleagues reported that administration of the peripherally-restricted CB_1_R inverse agonist, JD5037, reduced the intake of ethanol in wild-type mice; however, it was ineffective in whole-body CB_1_R- and GHS-R1a-null mice. Ethanol-consuming mice also had elevated levels of the eCB, anandamide, in stomach cells, and inhibiting peripheral CB_1_Rs with JD5037 blocked formation of the bioactive form of ghrelin, octanoyl-ghrelin. These results suggest that CB_1_Rs in stomach cells promote ethanol intake by a mechanism that includes controlling production of ghrelin. Next, a mouse gastric ghrelinoma cell line (MGN3-1)—which contains CB_1_Rs and enzymatic machinery for eCB metabolism—was used to identify mechanisms of CB_1_R-mediated ghrelin production. Inhibition of CB_1_Rs in MGN3-1 cells with JD5037 blocked formation of octanoyl-ghrelin by a mechanism that includes increased oxidative degradation of the ghrelin substrate, octanoyl-carnitine. Moreover, given expression of ghrelin receptors in the brain as well as vagal afferent neurons [82,94,95], the authors aimed to identify if the actions of JD5037 to reduce ethanol intake via changes in ghrelin signaling required gastric vagal afferent neurons. Both JD5037 and the CB_1_R inverse agonist, rimonabant, were ineffective at reducing ethanol intake in mice subjected to chemical ablation of sensory afferents by neonatal exposure to capsaicin. Interestingly, mice denervated by capsaicin displayed moderate increases in preference and intake of ethanol. Additionally, mice treated with JD5037 displayed no changes in ad-libitum intake of standard rodent chow under these specific conditions. Together, these studies provide evidence that CB_1_Rs in mouse stomach cells control intake and preference for ethanol by a mechanism that includes regulating production of ghrelin and indirect control of gut-brain vagal signaling. Future studies will be important to clarify if activating CB_1_Rs stimulates production of ghrelin by increasing conversion of octanoyl-carnitine to octanoyl-ghrelin, and if roles for these pathways extend beyond intake and preference for ethanol to other reinforcers, such as palatable food. In addition, the precise impact that CB_1_R-mediated control of ghrelin production has on vagal neurotransmission and associated firing rates remains to be determined.

Notably, ghrelin and CCK inversely affect vagal afferent neural activity, with ghrelin decreasing [94] and CCK increasing activity [6]. Accordingly, it is possible that eCB signaling at CB_1_Rs (*i*) on stomach cells that produce ghrelin [81] and (*ii*) on CCK-containing cells in the upper small-intestinal epithelium that inhibit CCK release [41] results in similar reductions in vagal afferent neural activity and increases in food intake. Moreover, these pathways may coordinate vagal afferent neural activity associated with feeding status and become imbalanced in diet-induced obesity. A direct test of these hypotheses, however, remains for future investigations.

## 4. Endocannabinoids and Gut-Brain Neurotransmission: Direct Mechanisms

In addition to indirect mechanisms, eCBs may also activate CB_1_Rs located on the afferent vagus nerve and directly impact gut-brain neurotransmission and food intake (see Figure 2). Indeed, a series of studies by Burdyga and colleagues suggest that expression of CB_1_Rs on rat gastric vagal afferent neurons is impacted by feeding status and gut-derived hormones. Immunoreactivity and mRNA for CB_1_Rs were identified in the rat and human nodose ganglia [83], and fasting for up to 48 h in rats was associated with time-dependent increases in their expression [83,84]. Refeeding after a 48 h fast led to reductions in expression of mRNA for CB_1_Rs in nodose ganglia by 2-hrs after reintroduction of food, an effect mimicked by administration of bioactive CCK-8 in fasted rats. In addition, administration of a CCK_A_ receptor antagonist blocked refeeding-induced reductions in expression of mRNA for CB_1_Rs in fasted rats, which suggests a key role for CCK in these processes. Similarly, administration of ghrelin (*i*) blocked refeeding-induced reductions in expression of mRNA for CB_1_Rs in nodose ganglia and (*ii*) blocked the actions of CCK-8 administration on expression of mRNA for CB_1_Rs in fasted rats [82,84]. These results highlight the opposing actions that gut-derived satiation (i.e., CCK) and hunger (i.e., ghrelin) signals have on expression of CB_1_Rs in rodent vagal afferent neurons.

Several studies suggest that expression of CB_1_Rs in the nodose ganglia is dysregulated in rodent models of diet-induced obesity. Immunoreactivity for CB_1_Rs was elevated in the nodose ganglia in Zucker or Sprague Dawley rats that were maintained on high-fats diet for 8 weeks when compared to lean controls [85]. Similarly, mRNA for CB_1_Rs was elevated in nodose ganglia in mice fed a high-fat diet for 12 weeks [86]. In addition, refeeding after a fast [85] or administration of CCK-8 in Wistar rats maintained on a high-fat diet both failed to reduce levels of immunoreactivity for CB_1_Rs in nodose ganglia [87]. Moreover, levels of mRNA for CB_1_Rs were elevated in the nucleus of the solitary tract in rats maintained on a high-fat and sugar diet [96]. Collectively, these studies suggest that CB_1_R expression in rodent vagal afferent neurons is controlled by feeding status, and their meal-related expression is dysregulated by chronic exposure to high-fat diets.

Roles in food intake for CB_1_Rs expressed in vagal afferent neurons are unclear; however, Elmquist and colleagues reported that genetic deletion of CB_1_Rs in the afferent and efferent vagus nerve had no impact on food intake, body weight, or energy expenditure in mice maintained on standard rodent chow or a high-fat diet [97]. These results suggest that CB_1_Rs on vagal afferent neurons may be sufficient to promote food intake but are not required in these processes. With regards to food intake, these findings are also in line with the transient nature of feeding suppression in rodents administered CB_1_R antagonists, which suggests that CB_1_Rs may not be required for the long-term maintenance of food intake [21]. Nonetheless, a series of important studies investigated the impact of activating CB_1_Rs on the neurochemical phenotype of associated neurons and the function of gastric vagal afferent neurons in mice [84]. Similar to CB_1_Rs, fasting was associated with time-dependent increases in expression of melanin-concentrating hormone 1 receptor (MCH1R) in the nodose ganglia of rats, albeit at later time-points when compared to CB_1_Rs. In contrast, fasting was associated with time-dependent reductions in expression of neuropeptide Y receptor type 2 (Y2Rs). Administration of CCK-8 reversed the effects of fasting by decreasing expression of CB_1_Rs and MCH1Rs, and increasing expression of Y2Rs. Notably, administration of the eCB, anandamide, dose-dependently reversed the effects of CCK-8 on expression of CB_1_Rs, MCH1Rs, and Y2Rs. Moreover, administration of a CB_1_R inverse agonist reduced expression of CB_1_Rs and increased expression of Y2Rs with no effect on expression of MCH1Rs in fasted rats. Together, these studies reveal distinct changes in the neurochemical composition of vagal afferents neurons in response to CB_1_R activation and inactivation, and suggest that CB_1_Rs may directly modulate activity of vagal afferent neurons in response to food-related signals released from the gut.

Elegant studies conducted by Christie and colleagues suggest that CB_1_Rs control mechanosensitivity of gastric vagal afferent neurons, which may be dysregulated in diet-induced obesity [86,88,89]. For these studies, a mouse in vitro electrophysiological preparation was utilized that consists of isolated stomach and esophagus with attached vagal fibers and measurement of vagal afferent neural function and mechanosensitivity (see for detailed protocol [98]). Application of methanandamide—a stable analog of anandamide —led to a biphasic effect on activity of vagal fibers in response to stretch, with low doses reducing responses to stretch and high doses increasing responses [88]. These effects were found only in tension sensitive fibers, but not those innervating gastric mucosa. In contrast to mice maintained on standard rodent chow, mice maintained for 12 weeks on a high-fat diet were only responsive to the inhibitory effects of methanandamide on gastric vagal afferent neural activity [86]. To identify receptor signaling pathways mediating these effects, methanandamide was co-incubated with a CB_1_R inverse agonist, a transient receptor potential vanilloid-1 channel (TRPV1) antagonist, a growth hormone secretagogue receptor (ghrelin receptor, GHSR) antagonist, or several inhibitors of distinct second messenger pathways (i.e., protein kinase A, protein kinase C, G-protein subunits Ga_io_ or Ga_q_). The biphasic effects of methanandamide on mechanosensitivity were both inhibited by CB_1_R and TRPV1 blockade in mice maintained on standard rodent chow. Furthermore, the excitatory effects of methanandamide may occur via a CB_1_R-mediated PKC-TRPV1 second messenger pathway, whereas the inhibitory effects may occur via CB_1_R-mediated release of ghrelin from the stomach and its actions on GHSRs on vagal afferent neurons. Together, these studies suggest that endocannabinoids differentially control afferent vagal activity depending upon dose by mechanisms that include distinct interactions between CB_1_Rs, TRPV1, and GHSR signaling pathways, which may become dysregulated in diet-induced obesity. Future studies will be important to identify physiological roles in food intake and obesity for CB_1_R signaling in distinct populations of gastric vagal afferent neurons (e.g., tension-responsive fibers versus those innervating the gastric mucosa). Moreover, it will be important to delineate how CB_1_Rs on sensory vagal terminals in the gut, nodose ganglia, and at terminals in the NTS may participate in distinct or common aspects of vagal afferent neurotransmission.

## 5. Endocannabinoids and Efferent Autonomic Neurotransmission

**Figure 3:**
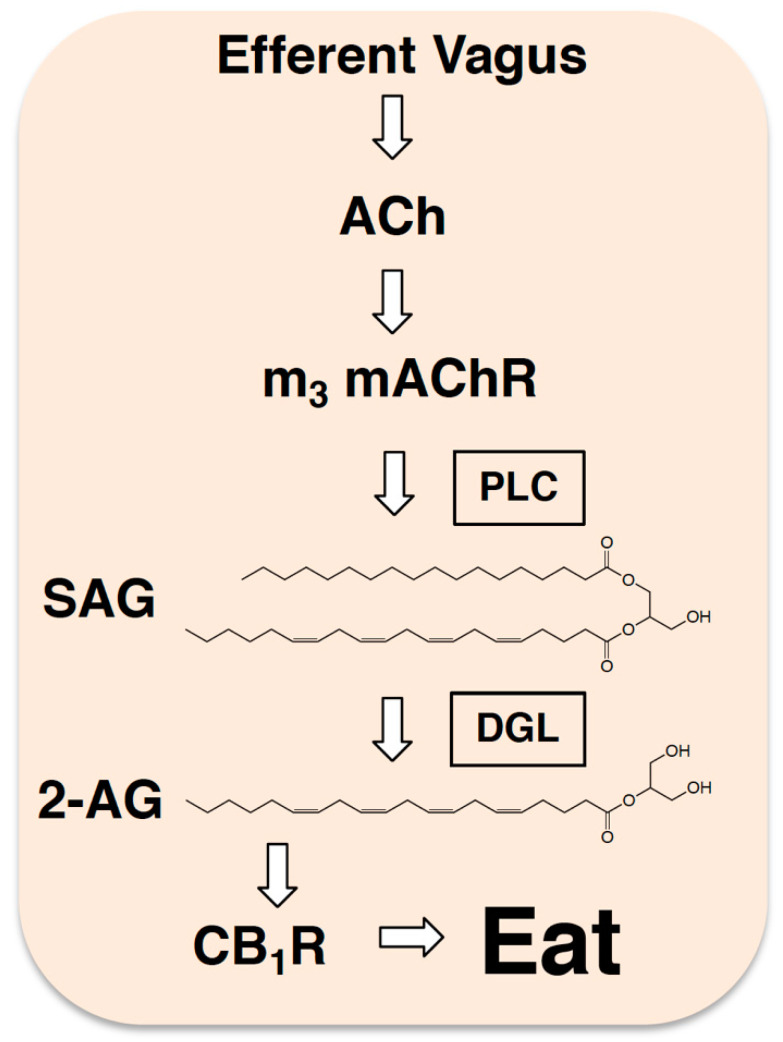
Efferent neurotransmission controls production of 2-AG in the gut. Studies suggest that during a fast, the efferent vagus nerve releases acetylcholine (ACh) into the lining of the small intestine, which in turn, activates local m3-subtype muscarinic acetylcholine receptors (m_3_ mAChRs) that trigger production of 2-AG (see [24]). This is thought to happen by a mechanism that includes activation of phospholipase C (PLC) and generation of the diacylglycerol 2-AG precursor, 1, stearoyl,2-arachidonoyl-*sn*-glycerol (SAG). SAG is subsequently hydrolyzed by diacylglycerol lipase (DGL) to ultimately form 2-AG, which activates local CB_1_Rs and promotes refeeding after a fast in rodents.

Fasting is associated with elevated levels of eCBs in the upper small-intestinal epithelium of rodents, and recent studies suggest that the efferent vagus nerve is required for these processes [22,24,25,40]. The efferent arm of the vagus nerve communicates parasympathetic neurotransmission from the brain to peripheral organs—including the gut—via cholinergic signaling pathways, and participates in a variety of motor functions and possibly food intake. For example, c-Fos expression in the myenteric and submucosal plexus in the rat proximal small intestine was induced by vagal nerve stimulation [99,100], and pharmacological blockade of peripheral muscarinic acetylcholine receptors (mAChRs) with atropine methyl nitrate inhibited both refeeding after a fast [101] and sham feeding of liquid diets in rats [102]. The specific receptor pathways involved in these processes are not fully elucidated. Recent investigations, however, suggest that cholinergic neurotransmission carried by the efferent vagus nerve controls biosynthesis of the eCB, 2-AG, in the proximal small-intestinal epithelium during a fast and participates in refeeding after a fast [24] (see Figure 3). For these studies, rats were fasted for up to 24 h, then levels of 2-AG and its precursor, 1, stearoyl, 2-arachidonoyl-*sn*-glycerol (SAG), were quantified in a variety of peripheral organs by liquid chromatography/tandem mass spectrometry. Levels of 2-AG and SAG were elevated in the upper small-intestinal epithelium by 24 h after fasting; however, no changes were found in stomach, ileum, colon, liver, pancreas, or spleen. This effect was specific for 2-AG, because levels of other common monoacylglycerols in the upper small-intestinal epithelium were unaffected (i.e., 16:0 MAG, 18:0 MAG, 18:1 MAG, 18:2 MAG). Moreover, levels of 2-AG were rapidly normalized by refeeding to levels similar to those in free-feeding animals, an effect mimicked by intra-duodenal infusions of equicaloric quantities of lipid, sucrose, or protein. These results highlight that production of intestinal 2-AG in fasting rats can be rapidly reduced upon refeeding in a manner that is not dependent on macronutrient content.

We next aimed to identify if efferent cholinergic activity is required for production of 2-AG in the intestinal epithelium [24]. Rats were given full diaphragmatic vagotomy and fasted for 24 h. When compared to control rats receiving a sham surgery, levels of 2-AG failed to increase in the small-intestinal epithelium after a 24 h fast, which suggests that the efferent vagus may be required for production of 2-AG. We next aimed to identify specific cholinergic receptors involved in vagal-mediated 2-AG production during a fast. The principal neurotransmitter released from the efferent vagus nerve is acetylcholine, which activates a variety of cholinergic receptor subtypes in the periphery, including mAChRs in the intestine [103]. Activation of mAChRs in the brain, including subtype-3 (m3) mAChRs, enhances eCB production that, in turn, participates in the control of synaptic plasticity via CB_1_Rs [104,105,106,107,108]. In addition, 2-AG in the brain is formed by a mechanism that includes phospholipase-C-dependent production of SAG, then conversion of SAG to 2-AG by diacylglycerol lipase [109,110,111]. Notably, m3 mAChRs are G_q_-protein coupled receptors that share overlapping downstream pathways as those responsible for biosynthesis of 2-AG, including activation of phospholipase-C and diacylglycerol lipase. Thus, we examined if mAChRs are required for fasting-induced production of 2-AG in the small-intestinal lining. Similar to brain, m3 mAChRs were expressed in the small-intestinal epithelium, and diacylglycerol lipase activity was required for biosynthesis of 2-AG. Systemic administration of a general mAChR antagonist (atropine) or intra-duodenal administration of a selective m3 mAChR antagonist both blocked fasting-induced rises in 2-AG in the small intestine. Moreover, pharmacological inhibition of peripheral CB_1_Rs and m3 mAChRs in the intestine equally reduced refeeding after a 24 h fast, with no additive effects when both inhibitors were co-administered. Collectively, these studies suggest that the efferent vagus nerve is recruited during a fast and participates in refeeding after a fast by activating m3 mAChRs in the intestine which, in turn, drives production of 2-AG and activation of local CB_1_Rs.

In addition to interactions between efferent parasympathetic neurotransmission and the eCB system, studies also suggest that efferent sympathetic neurotransmission is controlled by CB_1_Rs, which may impact food intake through a mechanism that requires the afferent vagus nerve [112]. Feeding suppression associated with a CB_1_R inverse agonist was abolished in mice that received (i) a peripheral β-adrenergic inhibitor, (ii) chemical ablation (capsaicin) of afferent sensory fibers, including afferent vagal fibers, and (iii) microinjections of the NMDA glutamatergic receptor antagonist, MK-801, into the nucleus of the solitary tract. Moreover, metabolic benefits of Roux-en-Y gastric bypass in mice were dependent on a mechanism that included interactions between CB_1_Rs and sympathetic neurotransmission [113]. Studies will be important to identify possible roles for CB_1_Rs in interactions between sympathetic and parasympathetic branches of the autonomic nervous system and their participation in control of food intake and energy metabolism.

## 6. Targeting the Peripheral ECB System for Treatment of Human Obesity

The pre-clinical studies in rodents discussed above suggest that the eCB system is an integral component of the gut-brain axis that controls food intake and becomes dysregulated in obesity. These investigations provide evidence of specific molecular and cellular mechanisms that underlie eCB-mediated gut-brain signaling, which may inform development of therapeutic strategies for the treatment of human obesity and related metabolic disorders. Indeed, human studies indicate that eCB levels are elevated in blood during consumption of palatable foods and in obesity [66,67,68,69,70,71,73,74,75,76,77,80,114]. CB_1_R antagonists were under development during the 2000s for the treatment of human obesity. In particular, rimonabant—a systemically acting CB_1_R antagonist/inverse agonist—showed clinical promise for its anti-obesity effects that included reductions in body weight, waist circumference, and levels of circulating triglycerides, and increases in levels of high-density lipoprotein) [115]. Unfortunately, rimonabant was associated with psychiatric side effects such as increased depression and anxiety, which precluded its approval by the Food and Drug Administration for the treatment of obesity in the Unites States [38]. These effects were likely a result of its ability to access the brain and disrupt cognitive functions. On the other hand, CB_1_R antagonists that are designed to have low brain penetrance display similar anti-obesity effects as their brain-penetrant counterparts and may be a useful therapeutic strategy for safe and effective treatment of obesity and related metabolic disorders [21,23,24,27,28,29,30,37].

## 7. Future Considerations

Collectively, these investigations provide evidence that the eCB system in the gastrointestinal tract is a key component of the gut–brain axis that controls food intake and becomes dysregulated in diet-induced obesity. Exciting new studies suggest important interactions between the eCB system and the gut microbiome [35,78,116,117,118,119,120,121]; however, future investigations will be important to identify molecular and cellular mechanisms in these interactions, and the impact on gut-brain signaling important for food intake and energy metabolism in health and metabolic disease. In addition, studies will be important to elucidate specific intracellular mechanisms that the eCB system recruits to control release of satiation peptides and other signaling molecules from the intestinal epithelium, including those involved in transduction of food-related signals to activation of vagal afferent neurons by “neuropods” [9,10]. It is clear that eCBs have indirect and direct actions on vagal afferent neural signaling; however, it is unclear how gut-brain eCB signaling interacts with brain reward circuits in control of food intake and reward. Moreover, it will be important to identify how gut-brain eCB signaling participates in discrete aspects of food intake and reward, including satiation and satiety versus appetition (i.e., post-oral positive feedback from nutrients that stimulates ingestion and flavor conditioning) [122].
